# Cellular Interaction Analysis Characterizing Immunosuppressive Microenvironment Functions in MM Tumorigenesis From Precursor Stages

**DOI:** 10.3389/fgene.2022.844604

**Published:** 2022-03-23

**Authors:** Zhenhao Liu, Siwen Zhang, Hong Li, Jiaojiao Guo, Dan Wu, Wen Zhou, Lu Xie

**Affiliations:** ^1^ Department of Hematology, Xiangya Hospital, Central South University, Changsha, China; ^2^ Key Laboratory of Carcinogenesis and Cancer Invasion, Ministry of Education, Key Laboratory of Carcinogenesis, National Health and Family Planning Commission, Cancer Research Institute, School of Basic Medical Science, Central South University, Changsha, China; ^3^ Shanghai-MOST Key Laboratory of Health and Disease Genomics, Institute for Genome and Bioinformatics, Shanghai Institute for Biomedical and Pharmaceutical Technologies, Shanghai, China; ^4^ Tongren Hospital, Shanghai Jiao Tong University School of Medicine, Shanghai, China; ^5^ Center for Biomedical Informatics, Shanghai Children’s Hospital, Shanghai Jiao Tong University, Shanghai, China; ^6^ Bioinformatics Center, National Clinical Research Centre for Geriatric Disorders, Department of Geriatrics, Xiangya Hospital, Central South University, Changsha, China

**Keywords:** single-cell RNA-seq, multiple myeloma, immune microenvironment, cell-cell interaction, immunoregulation

## Abstract

Cell–cell interaction event (CCEs) dysregulation may relate to the heterogeneity of the tumor microenvironment (TME) and would affect therapeutic responses and clinical outcomes. To reveal the alteration of the immune microenvironment in bone marrow from a healthy state to multiple myeloma (MM), scRNA-seq data of the four states, including healthy state normal bone marrow (NBM) and three disease states (MGUS, SMM, and MM), were collected for analysis. With immune microenvironment reconstruction, the cell types, including NK cells, CD8^+^ T cells, and CD4^+^ T cells, with a higher percentage in disease states were associated with prognosis of MM patients. Furthermore, CCEs were annotated and dysregulated CCEs were identified. The number of CCEs were significantly changed between disease states and NBM. The dysregulated CCEs participated in regulation of immune cell proliferation and immune response, such as MIF-TNFRSF14 interacted between early B cells and CD8^+^ T cells. Moreover, CCE genes related to drug response, including bortezomib and melphalan, provide candidate therapeutic markers for MM treatment. Furthermore, MM patients were separated into three risk groups based on the CCE prognostic signature. Immunoregulation-related differentiation and activation of CD4^+^ T cells corresponded to the progression status with moderate risk. These results provide a comprehensive understanding of the critical role of intercellular communication in the immune microenvironment over the evolution of premalignant MM, which is related to the tumorigenesis and progression of MM, which moreover, suggests a way of potential target selection for clinical intervention.

## Introduction

Multiple myeloma (MM) is a common, genetically heterogeneous, and incurable cancer (Palumbo and Anderson, 2011). MM is the second largest hematological malignancy (Hagen and Stiff, 2019), which is mainly characterized by the malignant proliferation of plasma cells in the bone marrow (BM). There are two precursor stages of MM, including monoclonal gammopathy of unknown significance (MGUS) and smoldering MM (SMM) (Rajkumar, 2019). The proportion of patients in the MGUS and SMM stages who develop into MM is about 1% and 10% each year (Dhodapkar, 2016). During patient progression from normal BM (NBM)–MGUS–SMM–MM, early immune changes are demonstrated (Zavidij et al., 2020). The tumor microenvironment (TME) can severely impair immunotherapy efficacy by repressing the immune system (Perrin et al., 2021). Systematically uncovering the alterations of the immune TME, especially intercellular communications in immunoregulation, may improve the efficacy of immunotherapy.

Recent studies on MM reveal the important role of immune TME. Compared with healthy control samples, MM patients were proved to have heterogeneous immune TME (Ledergor et al., 2018). Poorly characterized disease heterogeneity hampers early diagnosis of MM and treatment improvement. Natural killer (NK) cells were found increased in the precursor states of MM, associated with the changes of the chemokine receptors’ expression (Zavidij et al., 2020). Chemokine receptors are a important receptor family in cell-to-cell interaction of TME. Cell communication-related ligand and receptors, including VEGF, TNF, play crucial roles in the growth, survival, and dissemination of malignant plasma cells in patients of MM (Jasrotia et al., 2020). Further studies are needed to uncover the landscapes of cell interaction alteration from precursor states to MM, aiming to reveal the molecular mechanism between disease progression.

In this study, scRNA-seq data of samples in healthy and precursor disease states to MM, including NBM, MGUS, SMM, and MM, were integrated to reconstruct the immune microenvironment related to MM. The percentage of immune cell types, including NK, CD8^+^ T, and CD4^+^ T cells, were analyzed and associated with the prognosis of MM patients. Cell-to-cell communication events between immune cells were then annotated. The CCE changes between different pathological states were analyzed. The dysregulated CCEs were defined and selected in our work, their function annotated, and drug relation investigated when available. Disease state–specific CCE-based interaction networks were constructed. Finally, a cell interaction–based prognostic signature was constructed to stratify the MM patients. The changed cell interaction may result in the immunosuppressive microenvironment related to tumorigenesis and progression of MM. Cellular interaction genes could be candidate markers or drug targets for MM precision treatment. Our study provides a bioinformatics workflow of analyzing cell-to-cell interactions in scRNA-seq data for the interpretation of precision medicine research.

## Materials and Methods

### Data Collection

Aiming to reveal the microenvironment alterations associated with MM tumorigenesis, scRNA-seq data were collected from Gene Expression Omnibus (GEO). The GEO data set GSE124310 (Zavidij et al., 2020), including single-cell transcriptome profiles of samples from NBM, MGUS, SMM, and MM were downloaded. The sequencing libraries were constructed using the 10X genomics platform. Cellranger (v.2.0.1), the single-cell software suite from the 10X Genomics platform was used for alignment and counting analysis with the reference genome (hg38). The matrix generated by cellranger was downloaded. Further, bulk RNA-seq data for MM samples from MMRF were downloaded from the GDC data portal.

### Single Cell RNA-Seq Data Analysis

Seurat (Version 3.1.1) (Stuart et al., 2019) was mainly used for scRNA-seq data integration and downstream analysis. The quality of cells were then evaluated based on three metrics, cells with percent. mt < 20%, gene number >200 and <2500, and genes expressed in fewer than three cells were filtered. The data was integrated by *IntegrateData* function to eliminate the batch effect. Then, 30 principal components (PCs) were used for dimensional reduction and cell clustering. The resolution parameter was 0.5. Cluster specific markers were identified by *FindAllMarkers* and *FindMarkers* functions. The R package SingleR (Aran et al., 2019) was performed for cell type annotation.

### Cell–Cell Interaction Analysis

Aiming to reveal the CCEs among different cell types and compare the difference between MM and the precursor stages, cellphoneDB (Version 2.1.5) (Efremova et al., 2020) was applied. The interaction pairs with *p*-value < .05 were reserved as significant CCEs. Fisher’s exact test was performed to identify CCE enriched cell types. Here, to define dysregulated CCEs in our work, according to the annotation in cellphoneDB, the expression of interacting pairs were calculated by formula 1. Then, we defined the CCE fold change (formula 2), and the CCE with the absolute value of fold change >0.25 refers to this CCE being differentially interacted and dysregulated in two pathological states. Further, the genes in CCE were separated as ligand and receptor for further functional investigation. The genes annotated as the “True” receptor in the interacting pair were set as receptors interacted in the CCE. The “False” one was set as ligand. Ligand-derived cell types were treated as regulatory cells (source cell types), and the receptor-derived cell types as regulated cells (target cell types). Functional enrichment analysis was performed to reveal the alteration of biological processes and pathways in target cells.

$$EXP_{CCE}=Mean\left(Receptor,Ligand\right)$$ (1)

$$FoldChange_{CCE}=\frac{EXP_{CCE\text{ in State}1}\;-\;EXP_{CCE\text{ in State}2}}{Minium\left(EXP_{CCE\text{ in State}1},\;\;EXP_{CCE\text{ in State}2}\right)}$$ (2)

If $FoldChange_{CCE}\;>\;0.25\;or\;EXP_{CCE\text{ in Group}2}=\;0$ , the CCE is upregulated in State1. If $FoldChange_{CCE}\;<\;-0.25orEXP_{CCE\text{ in Group}1}=\;0$ , the CCE is downregulated in State1.

### Immune Cell Infiltration in Microenvironment

CIBERSORT (Newman et al., 2015) and ImmuCellAI (Miao et al., 2020) were performed to estimate immune infiltrates with transcriptome profiles of MM patients. MM samples from MMRF with tissue source as “Primary Blood Derived Cancer—Bone Marrow” were analyzed in this study. There are 22 infiltrated immune cell types predicted by CIBERSORT. Whereas, by ImmuCellAI, 24 immune cell types, mainly including 18 T cell subtypes, were predicted. The patients are split into two groups according to the infiltrated proportion of the immune cells. The number of patients in the smaller group should be greater than 20% of all patients. The Kaplan–Meier survival plot (KM-plot) was applied to compare the two cohorts, and the log-rank *p*-value are calculated. The infiltrated immune cells with log-rank *p*-value < .05 were associated with MM patients’ progression. Further, univariable Cox regression analysis was performed to identified progression-related infiltrated immune cell types, too.

### Cancer Drug Response Prediction

The Cancer Treatment Response gene signature DataBase (CTR-DB) was used for cancer drug response prediction. Genes involved in cellular communications were used as input. The AUC >0.7 and the AUC-adjusted *p* value <.05 were set as the thresholds for genes that can be used for drug response prediction. The expression of genes in the response and nonresponse groups were shown with a box plot. The ROC was also plotted.

### CCE-Based Prognosis Signature Construction

The genes function in cell interactions were used to constructed CCE-based prognosis signature. According to the results of univariable Cox regression analysis, genes with *p*-value < .05 were identified as significance associated with MM progression (overall survival, OS). Aiming to establish a robust prognostic signature, the transcriptome profiles of 763 samples in MMRF were separated as a training set (457 samples, about 60%) and test set (306 samples, about 40%). Furthermore, multivariable Cox regression analyses were performed with significant progression-related genes in the training set. The Akaike information criterion (AIC) statistic was used to select a model with function *step* in R packages stats. There are seven CCE genes retained in this study. The risk score was constructed based on the gene expression and the corresponding regression coefficients as follows:

$$RiskScore=\overset{7}{\underset{i=1}{\sum }}\beta_{i}*Gene_{i}$$ (3)

$\beta_{\text{i}}$ denotes the coefficient of $Ge{\text{ne}}_{\text{i}}$ and $Ge{\text{ne}}_{\text{i}}$ represents the *i*th marker gene in the prognostic model. To graphically exhibit the prognostic outcomes, samples were separated into three groups, including the high, moderate, and low risk groups. KM survival curves were generated then. The signature was validated in the test set.

### Statistical Analysis and Functional Enrichment Analysis

Functional enrichment analysis was performed with genes by clusterProfiler (Version 3.10.1) (Yu et al., 2012) in R. Enriched terms were kept with adjusted *p*-value <.05. Protein–protein interactions (PPIs) were annotated by STRING database (Version 11.0) (Szklarczyk et al., 2019). PPIs with a combined score ≥0.7 were reserved for next step analysis. Cytoscape (Version 3.7.2) (Shannon et al., 2003) was used to construct the CCE-based gene interaction network. Gene set enrichment analysis (GSEA, Version 4.1.0) was performed to identify enriched terms in different risk groups. All the statistical analyses in this study were calculated in R (Version 4.0.3) and Python (Version 3.7.7). Figures were plotted by the corresponding R package or by ggplot2 (Version 3.1.1) in R.

## Results

### Immune Microenvironment Reconstruction Based on scRNA-Seq

**FIGURE 1:**
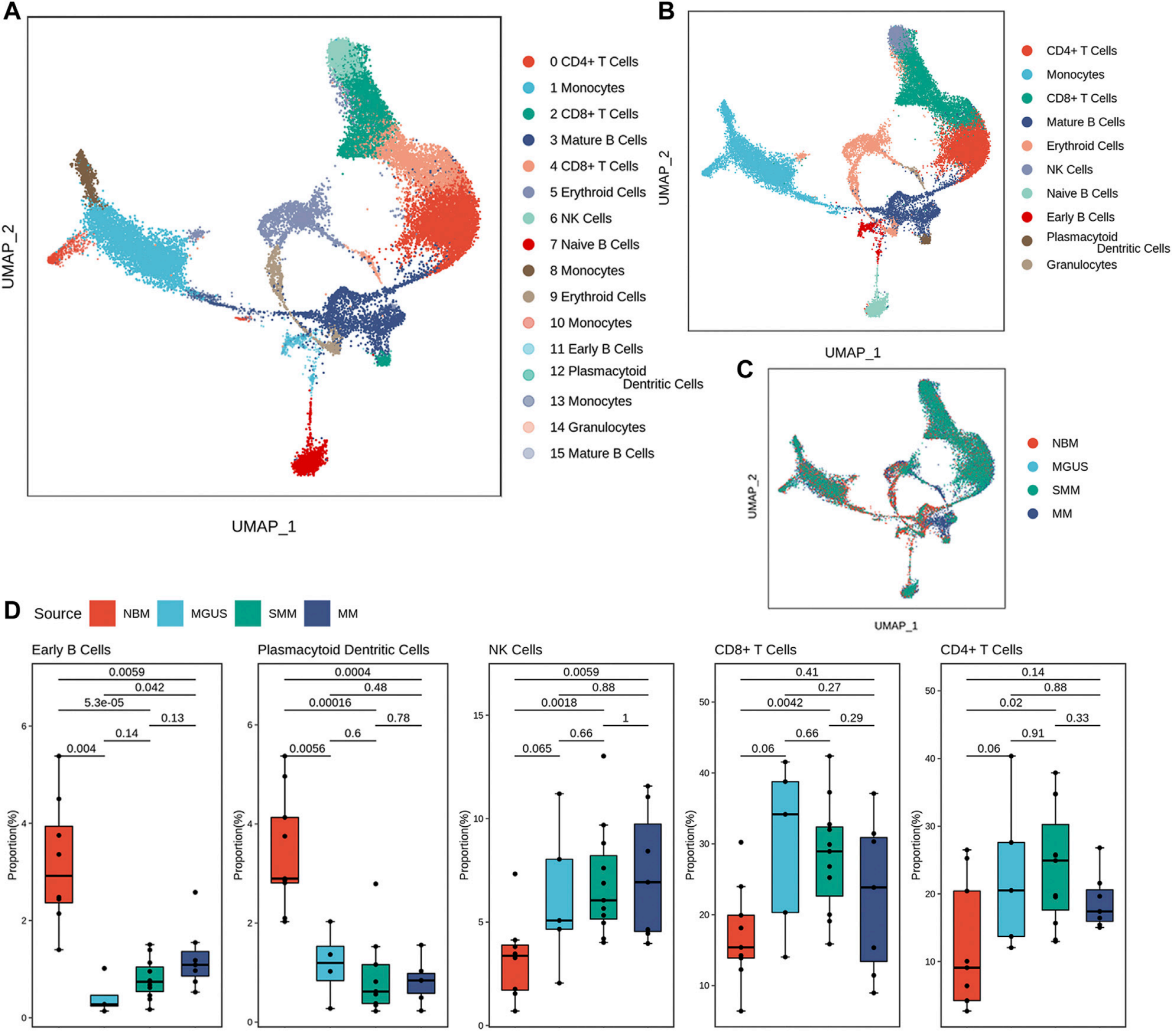
Diverse cell types in MM and precursor stages delineated by single-cell RNA-seq analysis. The UMAP plot demonstrates cell types **(A)**, main cell subtypes **(B)**, and cells’ source in the clusters **(C)**. Boxplot of the cells with significant proportion change in the four stages **(D)**.

After quality control, there are about 25,000 cells from 32 samples reserved for immune microenvironment (IME) reconstruction. According to the expression patterns, the cells were clustered into 16 cell groups (Supplementary Tables S1, S2). There are 10 cell subtypes (Figures 1A–C). The proportion of cell subtypes in the samples were compared to identify the difference in immune environment in the four states (Figure 1D). Early B cells and plasmacytoid dendritic cells had significantly high proportions in NBM compared with the disease status (Wilcox test, *p*-value < .05). The early B cells seemed to be gradient increasing in MGUS-SMM-MM while NK, CD4^+^ T, and CD8^+^ T cells were found to have significantly higher proportion in disease status than in NBM. The CD8^+^ T cells have a higher proportion in MGUS samples, higher than SMM and MM, showing a gradual downward trend. The median proportion of CD8+T cells in MM was higher than that in NBM.

### Infiltrated Immune Cell Proportion Changes Related to MM Progression

**FIGURE 2:**
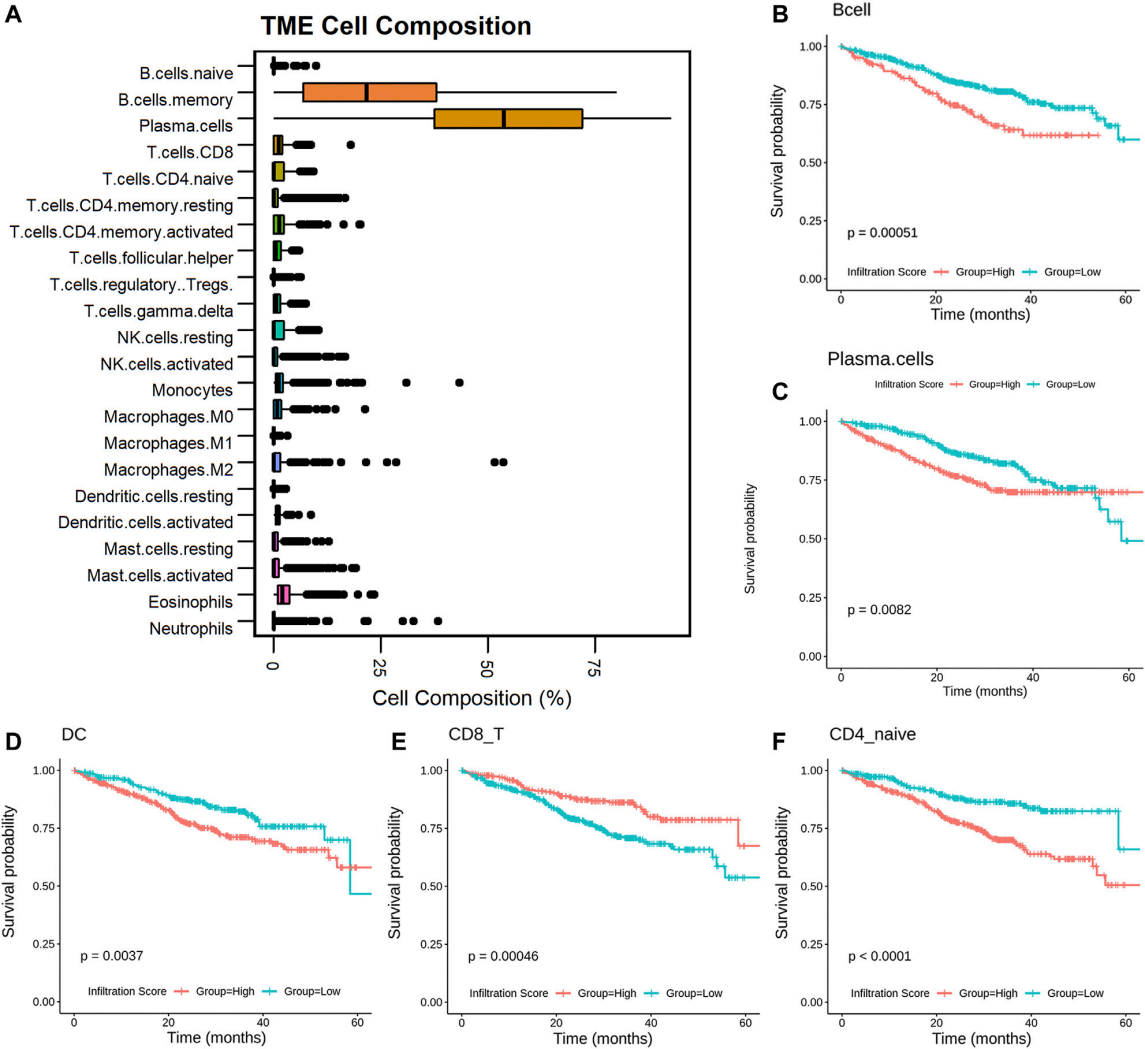
Infiltrated immune cells in MM associated with patients’ prognosis. Boxplot of the infiltrated immune cells **(A)**; KM-plot of the cells with infiltrated score predicted by CIBERSORT and ImmuCellAI **(B–F)**.

To reveal the role of proportionally changed immune cells in MM IME, the infiltrated immune cells of MM samples were estimated with the transcriptome profiles. There are 22 immune cell types predicted by CIBERSORT (Figure 2A) and 24 immune cell types predicted by ImmuCellAI. The plasma cells and memory B cells were found to have higher proportions in MM tissues, consistent with MM being a bone marrow plasma cell malignancy disease. The infiltration of 10 cell types resulted in the CIBERSORT prediction, including plasma, naive CD4^+^ T, and activated NK cells, which are significantly related to the prognosis (Figure 2C, Supplementary Figure S2). Among the infiltrated immune cell types predicted by ImmuCellAI, 14 cell types, mainly T cell subtypes, are significantly related to the prognosis (Figures 2B,D–F, Supplementary Figure S3, Supplementary Table S3). The cell types with different proportions in MM precursor states, including B, plasma, dendritic, CD8+T, naive CD4+T, and activated NK cells, are significantly related to the prognosis (*p*-value <.05). Among them, MM patients with the high infiltrating proportion of CD8^+^ T cells have a higher survival probability (*p*-value < .01), which is consistent with CD8^+^ T cells participating in cellular immunity to eliminate tumor cells and slow down the development of the disease. This suggests that alternations in the immune microenvironment play an important role in the occurrence and development of diseases and are related to the prognosis of patients.

### Immunoregulation Alteration in MM and Precursor Related to Tumorigenesis and Drug Response

**FIGURE 3:**
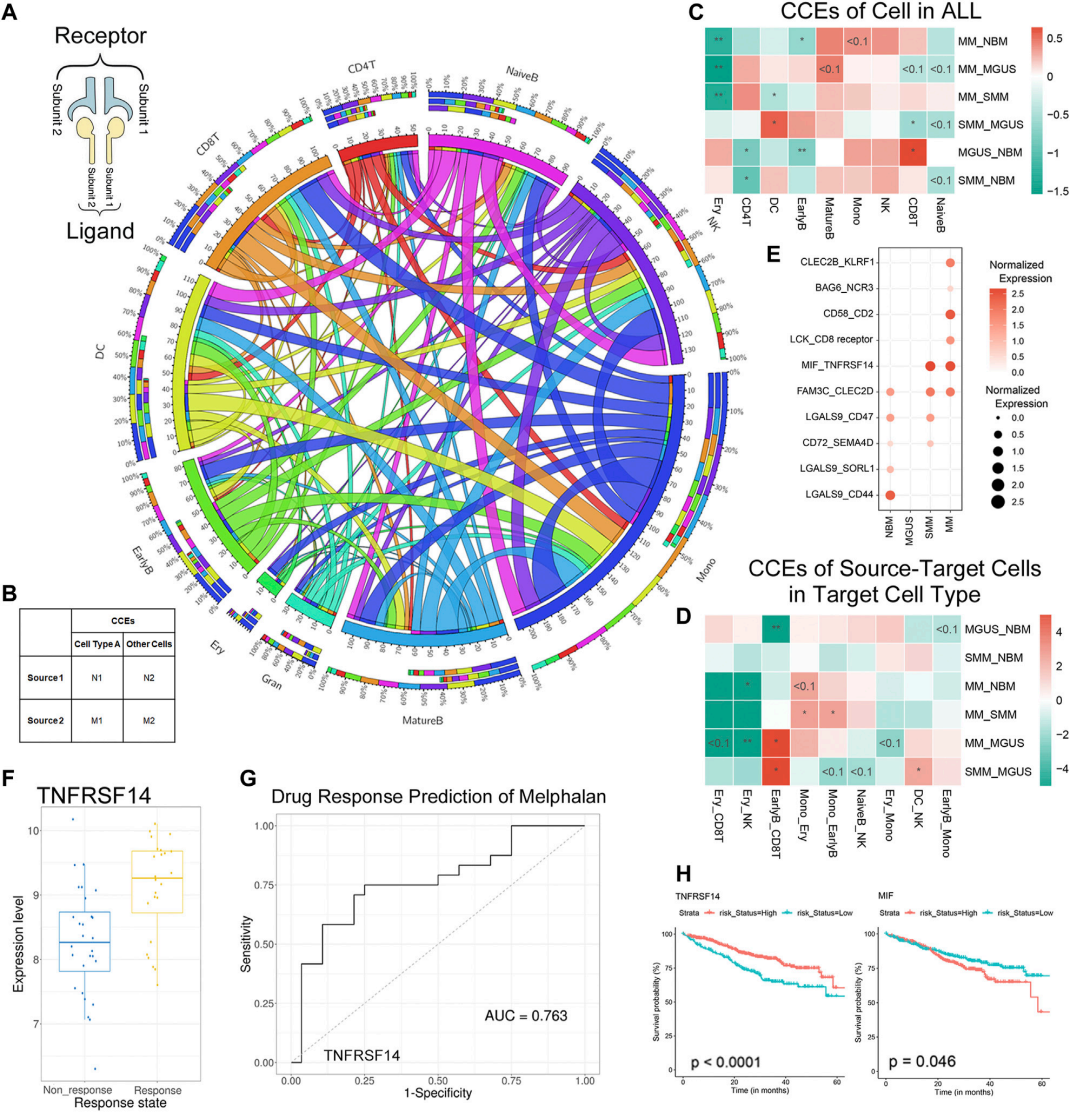
CCEs in MM and precursor stages annotated with CellPhoneDB. The Circos plot for the CCEs of immune cell interaction in MM TME **(A)**: the outside and inside circles represent the percentage and the count of CCEs, separately. The three-tiered ring from outside in represents the total CCEs of this cell type, the CCEs when the cells are regulated cell types, and the CCEs when the cells as regulatory cells. The input for Fisher’s exact test in the analysis **(B)**. The results of Fisher’s exact test when we calculated the CCEs of each cell types [color bar means log2 (odds ratio)] **(C)**. The results of CCEs in target cells when the target cell interacted with another cell type **(D)**. Dysregulated CCEs interacted in EarlyB-CD8T **(E)**. The boxplot for TNFRSNF14 in the response and nonresponse groups of melphalan **(F)** and the ROC for drug response prediction of melphalan **(G)** in CTR-DB; The KM-plot of TNFRSNF14 and MIF in GDC MMRF **(H)**.

Aiming to reveal the functional roles of immune cells in disease progression, CCEs between immune cells were annotated. There are 330 significant interaction events in NBM, 349 in MGUS, 372 in SMM, and 477 in MM (Figure 3A, Supplementary Figure S4, Supplementary Table S4). Monocytes were found with more CCEs in the immune microenvironment. To clarify whether there are significant differences in the number of interaction events under different interaction conditions, a contingency table is set up to perform Fisher’s exact test for data involving the number of CCEs and the number of other CCEs under different pathological conditions (Figure 3B).

First, we compared the cell interaction events of different cell types in the IME. The CD8^+^ T cells were with more CCEs in MGUS (Figure 3C) than in NBM (*p*-value < .05), SMM (*p*-value < .05), and MM (*p*-value < .1). It is consistent with the high proportion of CD8^+^ T cells (higher median) in the MGUS samples. Although the difference of the proportion of CD8^+^ T cells in the three pathological states are not at a significant level, there is a significant decrease of CCEs. The cell interaction event alterations may relate to the cell ratio changes. For CD4^+^ T cells and NK cells with higher cell proportion in pathological states, no significantly more CCEs were detected. DC cells were found with more CCEs in SMM, significantly more than MGUS and MM (*p*-value < .05).

Next, we analyzed the CCE alteration of the cells treated as the target cell type. When CD8^+^ T cells were targeted by early B cells (EarlyB_CD8T), CCEs in MM, SMM, and NBM are significantly more than MGUS (Figure 3D). Dysregulated CCEs were identified (refer to the methods section) then. MIF-TNFRSF14 was differentially interacted both in SMM and MM (Figure 3E), whereas elevated expression of MIF, a pro-inflammatory cytokine (Alibashe-Ahmed et al., 2019) and an oncogene (Yao et al., 2021), was associated with stronger suppression of T-cell proliferation (Zhang et al., 2017). The TNFRSF14 gene encodes a member of the tumor necrosis factor (TNF) receptor superfamily, which plays a role in the signal transduction pathway that activates inflammatory and inhibitory T-cell immune response. TNFRSF14 was identified as a marker for drug response prediction of melphalan in melanoma (Figures 3F,G, Supplementary Table S5) (Liu et al., 2021), and melphalan was used as the first line of therapy for MM patients in MMRF. The expression of MIF and TNFRSF14 were found associated with MM patients’ progression from MMRF (KM-plot, log-rank *p*-value < .05, Figure 3H). Therefore, the dysregulated CCE MIF-TNFRSF14 may imply a drug response mechanism.

Furthermore, the CCEs, including LCK_CD8 receptor and CD58_CD2, were found differentially interacted in MM, specifically. LCK is a proto-oncogene, a member of the Src family of protein tyrosine kinases (PTK), and the protein encoded by it is a key signal molecule for the selection and maturation of developing T cells. CD2 interacts with the lymphocyte function-related antigen CD58 (LFA-3), participating in mediation of adhesion between T cells and other cell types. CD2 is related to the triggering of T cells, and its cytoplasmic domain is related to signal transduction functions. Similarly, there are specific cell interaction events in the interaction of other cell types (Supplementary Figure S5).

### Disease State–Specific Interaction Network Construction Based on CCE

**FIGURE 4:**
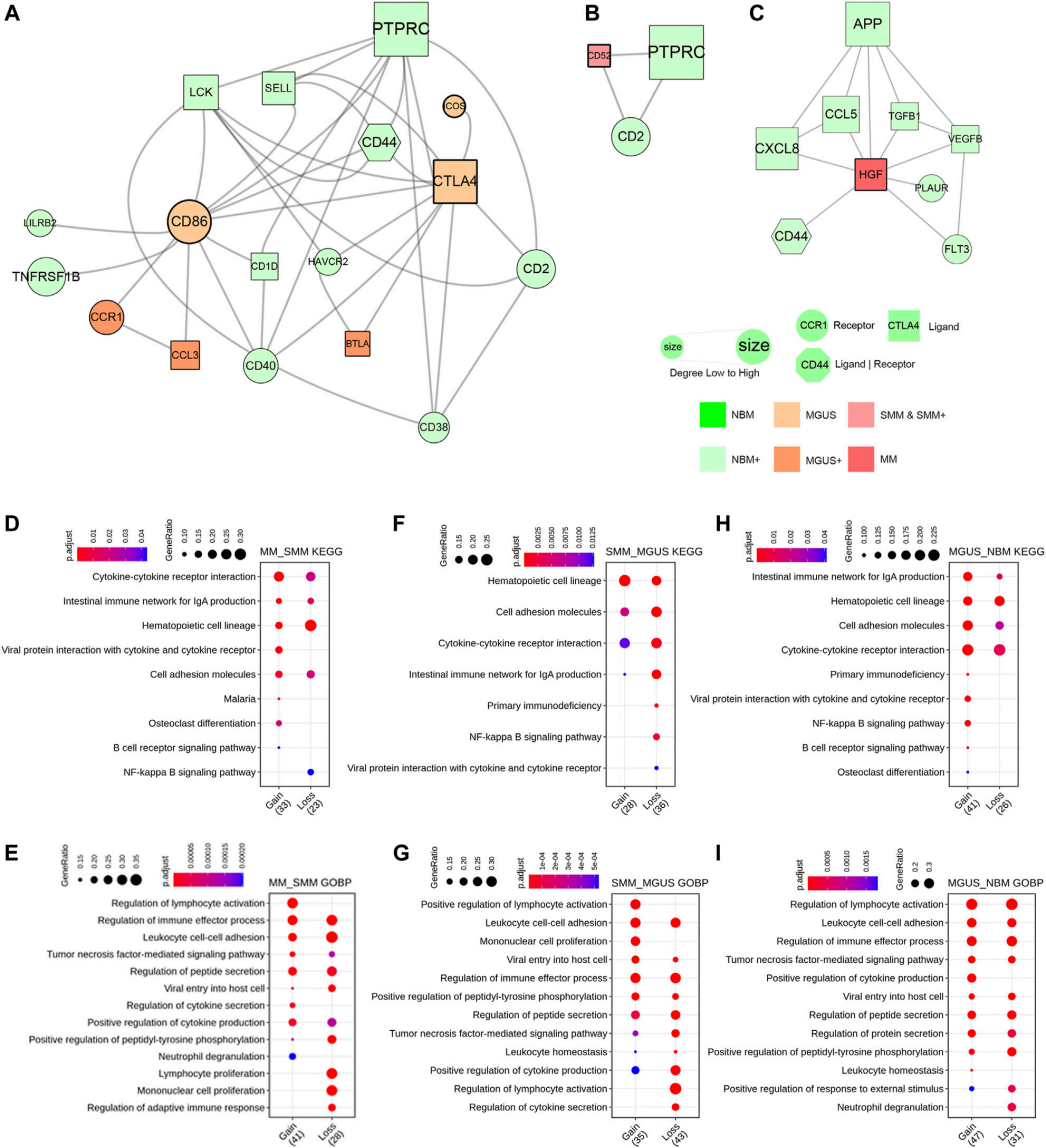
PPI network constructed with key ligand and receptor genes of CCEs. The MGUS-specific network **(A)**. The SMM-specific network **(B)**. The MM-specific network **(C)**. Enriched GO BP terms and KEGG pathways as gain or loss functions in comparison of MM to SMM **(D,E)**, SMM to MGUS **(F,G)**, and MGUS to NBM **(H,I)**.

The receptor and ligand genes involved in cell interactions in the IME under different pathophysiological conditions were integrated to construct the progression-related gene interaction network of NBM–MGUS–SMM–MM (Supplementary Figures S6–S8). A total of 115 receptor or ligand genes are used to construct a PPI network. Pathological state–specific ligand and receptor genes and associated genes were extracted to show the key interaction relationships while in MGUS, CTLA4 and CD86 were uniquely identified and with a higher degree in the network (Figure 4A). Binding of the CD86 encoding protein with cytotoxic T-lymphocyte-associated protein 4 (CTLA4) negatively regulates T-cell activation and diminishes the immune response (Tekguc et al., 2021). CD86 is involved in the regulation of B cell function, playing a role in regulating the level of IgG produced (Lanier et al., 1995). CD52, an approved nontherapeutic target for MM (Touzeau et al., 2017), is the unique gene in SMM immunoregulation (Figure 4B). HGF is the unique hub gene in the immune-interaction network of MM (Figure 4C). The HGF, which can regulate cell growth, cell motility, and morphogenesis in a variety of cell and tissue types, plays an important role in angiogenesis, tumor formation, and tissue regeneration.

To clarify the functional alteration related to pathological state gradient change in NBM–MGUS–SMM–MM, the receptor genes in differentially interacted CCEs were functionally annotated and enriched (Figures 4D–I, Supplementary Figure S9). In the comparison between MM and SMM, the receptor genes in the upregulated CCEs are significantly enriched in osteoclast differentiation, B cell receptor signaling pathway, and lymphocyte activation. Compared with the upregulated CCE in MGUS, SMM has a higher enrichment ratio in the hematopoietic cell lineage, and is significantly enriched in positive regulation of lymphocyte activation and monocyte proliferation. Compared with NBM, MGUS is significantly enriched in the NF-kappa B signaling pathway, B cell receptor signaling pathway, viral protein and cytokine, and its receptor interaction. The osteoclast differentiation pathway is significantly enriched with a higher gene ratio in MM than in SMM, MGUS, and NBM. This is consistent with MM patients with osteolytic changes, such as bone pain, osteoporosis, pathological fractures, and other pathological symptoms. The NF-kappa B signaling pathway has a higher gene ratio in MGUS (NBM < MGUS > SMM > MM). It plays an important role in the regulation of immune responses, such as infection, and its dysfunction has an important relationship with the occurrence of diseases, such as inflammation and cancer (Hoesel and Schmid, 2013; Vrábel et al., 2019).

### CCE-Based Prognosis Signature Construction

OS-related genes in MM patients were identified by univariate Cox regression analysis first. There are 3153 genes (FDR <0.05) reserved for functional annotation. The significantly enriched pathways and terms (Supplementary Figure S10), including cell cycle, DNA replication, mismatch repair, and DNA replication, were associated with tumorigenesis and progression of cancers. Genome instability increasing the tendency of genome changes is a sign of cancer, including MM (Alagpulinsa et al., 2020).

**FIGURE 5:**
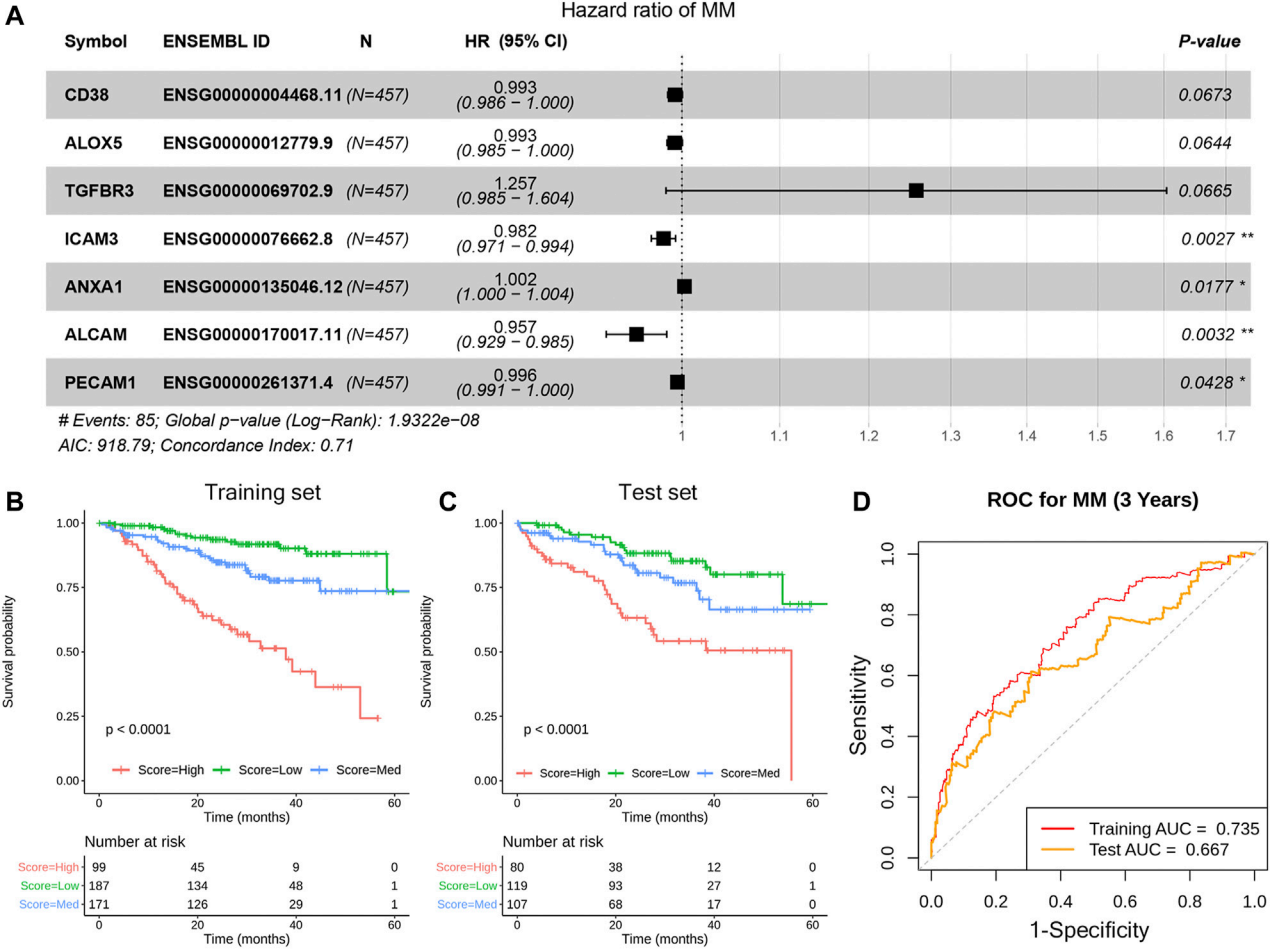
Construction of the prognosis model based on the CCE genes in TME. The forest plot of the seven genes in the model **(A)**, KM estimates of OS of MM patients in the training data set **(B)** and in the test data set **(C).** Based on the seven-gene signature, patients were divided into three risk groups according to risk score; the receiver operating characteristic (ROC) curve for OS survival predictions for the signature in training set and test set **(D)**.

The ligand and receptor genes significantly associated with MM progression remained. The OS-related CCE genes were used as input features to construct the model. After feature selection, seven genes (CD38/ALOX5/TGFBR3/ICAM3/ANXA1/ALCAM/PECAM1) were finally involved in MM prognostic model construction (Figure 5A). According to the predicted scoring of the model, patients can be divided into three groups, including high, low, and medium risk groups. The OS time of patients in the three risk groups is significantly different (median OS time is 18.43, 26.17, and 30.00 months, *p*-value <.001) (Figure 5B). Whereas, in the test set, the prognosis of the three groups of patients was significantly different (*p*-value < .01) (Figure 5C). Assessment of model accuracy, 3-year AUC for the training set was 0.735 and the value for the test set was 0.667 (Figure 5D).

**FIGURE 6:**
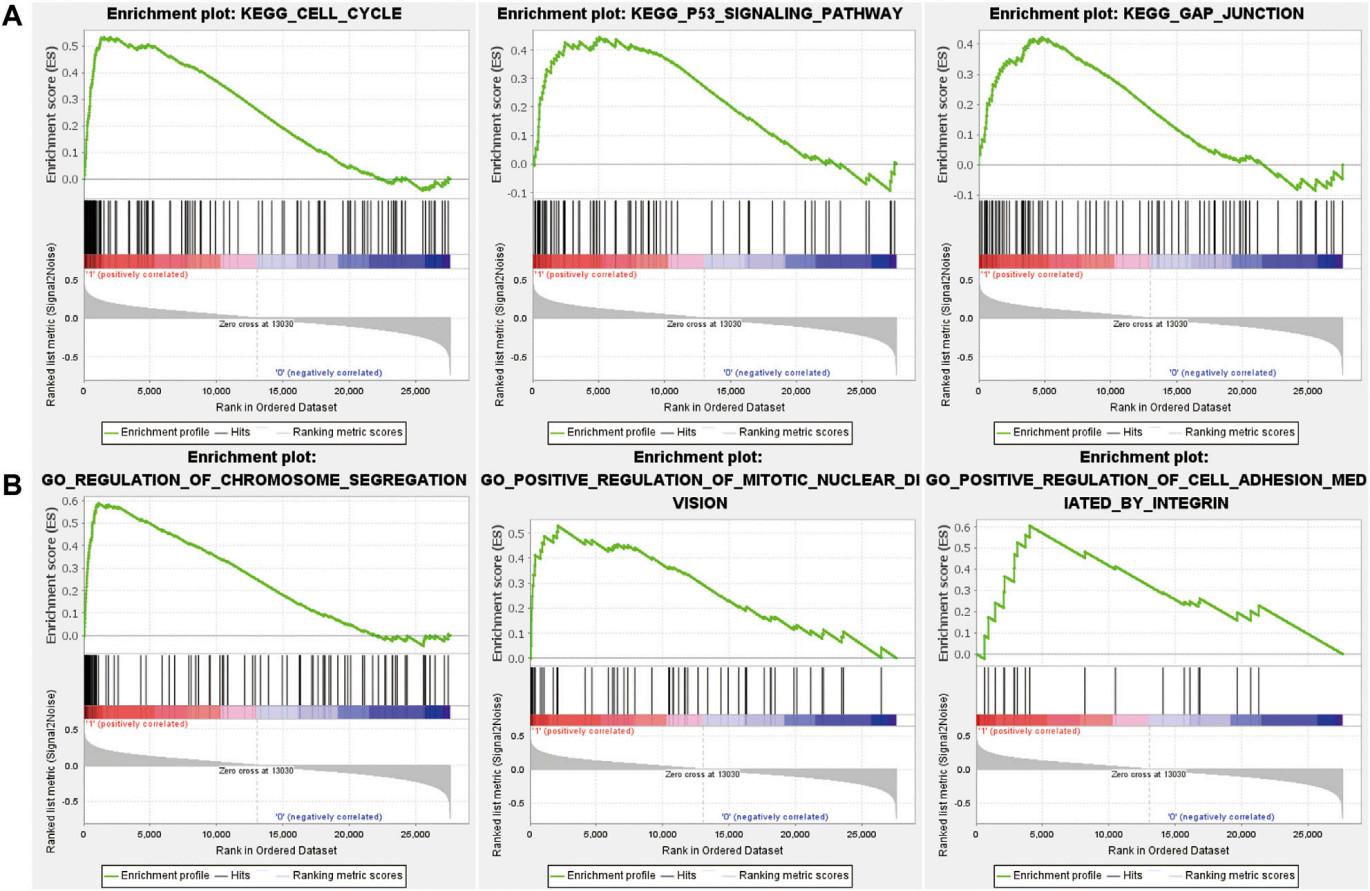
The results of GSEA analysis for comparison of high and low risk groups. Enriched KEGG pathways **(A)** and GO BP terms **(B)** in the high risk group.

GSEA analysis was performed on the three risk groups. Compared with the medium and low risk groups, the pathways including cell cycle and P53 signal pathway were significantly enriched in the high-risk group (Figure 6). The enriched pathways are similar to prognostic-related pathways. The NOTCH signaling pathway and complement and coagulation cascades signaling pathway were with higher enrichment score in the medium risk group than in the low risk group (Supplementary Figure S11). In addition, the activation and differentiation of CD4^+^ T cells and the differentiation of T-Helper 2 cells were significantly enriched in MM, too. The activation of the Notch signaling pathway affects the biological functions of myeloma cells in MM and promotes the reprogramming of stromal cells in the BM, supporting the growth and survival of tumor cells (Colombo et al., 2020). CD4^+^ T cells in MM can induce effective antitumor immune responses by interacting with antigen-presenting cells in the tumor microenvironment (Haabeth et al., 2020).

## Discussion

By profiling transcriptomics data of thousands of cells, scRNA-seq make it easy to study the cellular heterogeneity of the TME and the cellular communication alteration. Cellular communication is critical to coordinating diverse biological processes, such as development, differentiation, programmed cell death, and inflammation (Efremova et al., 2020; Jin et al., 2021). This study aims to investigate how the context-dependent crosstalk of different cell types enables physiological processes to proceed from precursor states to MM.

Currently, in our study, the transcriptional level sequencing data of immune cells from precursor states and MM were collected and analyzed. The study on cell communication dysregulation in the IME provides a new perspective for understanding the pathogenesis development. Cells, including early B, NK, CD8^+^ T, and CD4^+^ T cells, had different proportions from samples in disease states compared with NBM. The changed cell types participated in cellular immunity and immunoregulation of IME and were associated with MM progression. We identified differentially interacted cell types then. CD8^+^ T cells in MGUS had significantly more CCEs than in NBM, SMM, and MM, whereas the proportion of CD8^+^ T cells in disease states was not significantly different. Furthermore, the ligand-receptor interacting pair MIF-TNFRSF14 were identified interacted between early B and CD8^+^ T cells in SMM and MM. The expression of MIF was associated with stronger suppression of T-cell proliferation (Zhang et al., 2017). MIF induces the expression of CD84, which is a regulator of the immunosuppressive microenvironment in MM (Lewinsky et al., 2021). The TNFRSF14 is the marker for the drug response prediction of melphalan (Liu et al., 2021), which was used in the first line of therapy of MM (Buda et al., 2021). Thus, the dysregulated CCEs participating in IME regulation in the tumorigenesis of MM may proceed to the immune cell proportion change.

Furthermore, the disease state–specific immune interaction network was extracted to illustrate the mechanism of IME dysregulation. The unique hub gene HGF is associated with MM-induced bone disease by promoting osteoclast formation (Tsubaki et al., 2020). Furthermore, Met and NF-κB inhibitors, including bortezomib (BTZ), which is usually used for MM treatment, may also potentially mitigate MM-induced bone disease in patients expressing high levels of HGF by inhibiting osteoclast formation (Tsubaki et al., 2020). In summary, the different interacted CCEs were associated with disease development and might affect therapeutic responses and clinical outcomes of MM. A seven-gene MM prognosis prediction signature based on dysregulated CCE was constructed, which can be applied successfully for prognostic stratification in MM. The model exhibits good enough prediction ability. This suggests again the important role of cellular interaction in the development of MM. Future studies are needed to explore the precision treatment of MM patients stratified by the cellular interaction signature to improve the prognosis.

In conclusion, our comprehensive characterization of cells at the single level from different states from NBM–MGUS–SMM–MM revealed the cell composition nature and cellular communication pattern in the IME. Alteration of cellular communications between immune cell types were associated with the disease phenotype and clinical behavior. It may be indicative of surveillance for the alteration from NBM to MM. The IME in precursor states may accelerate tumorigenesis of MM. The genes involved in cellular communication such as TNFRSF14 and HGF related to drug response might serve as therapeutic markers in MM.

## Data Availability

The original contributions presented in the study are included in the article/Supplementary Material, further inquiries can be directed to the corresponding authors.
