# Supplementary material for: Cellular Interaction Analysis Characterizing Immunosuppressive Microenvironment Functions in MM Tumorigenesis From Precursor Stages
Source: Front Genet. 2022 Mar 23;13:844604. doi: 10.3389/fgene.2022.844604 (PMC8984155; doi:10.3389/fgene.2022.844604)
Supplement: Supplementary file 1 [file DataSheet1.PDF]

Figure S1.

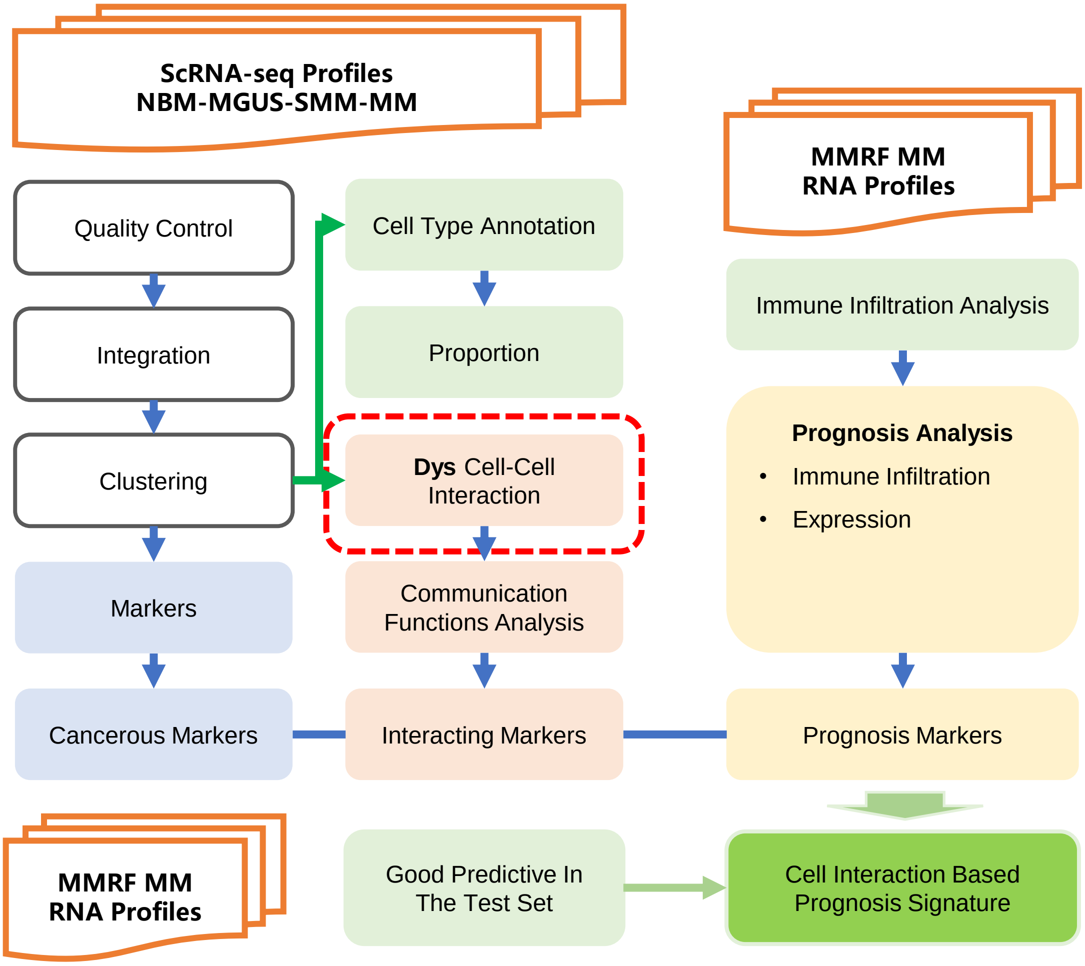

Figure S1. The workflow for this study.

Figure S2.

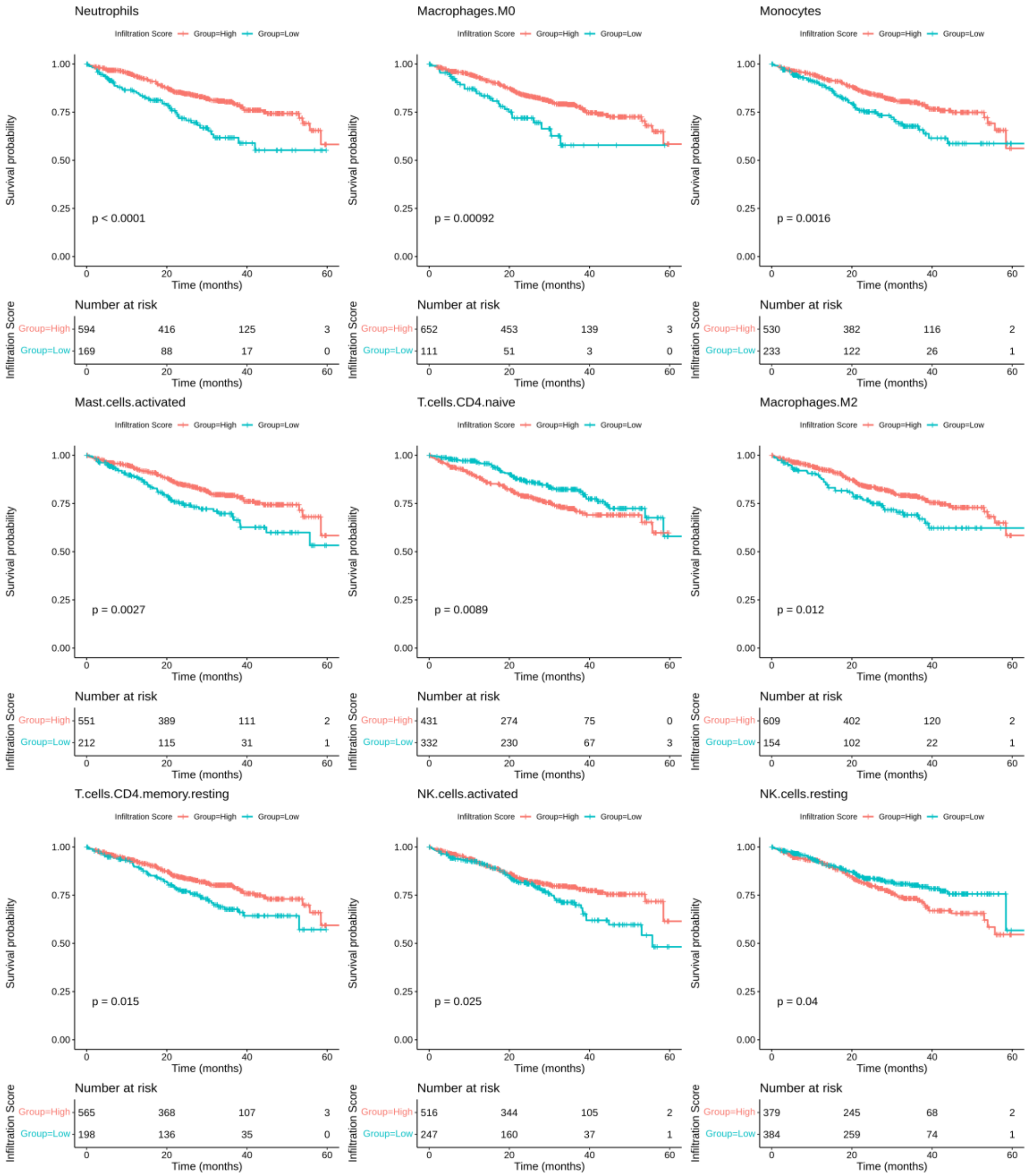

Figure S2. The KM-plot of CIBERSORT predicted infiltrated immune cells in GDC MMRF.

Figure S3.

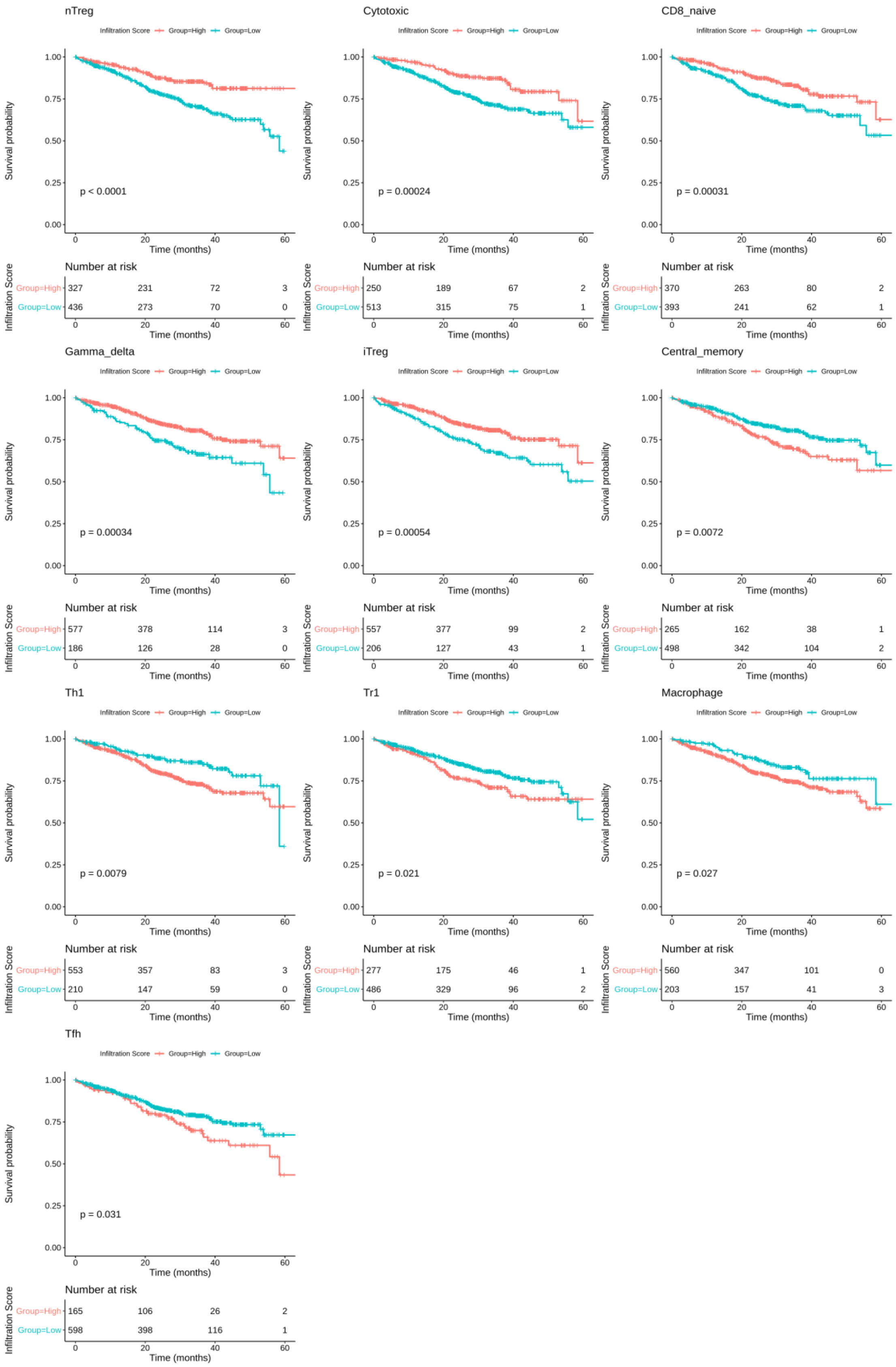

Figure S3. The KM-plot of ImmuCellAI predicted infiltrated immune cells in GDC MMRF.

Figure S4.

NBM

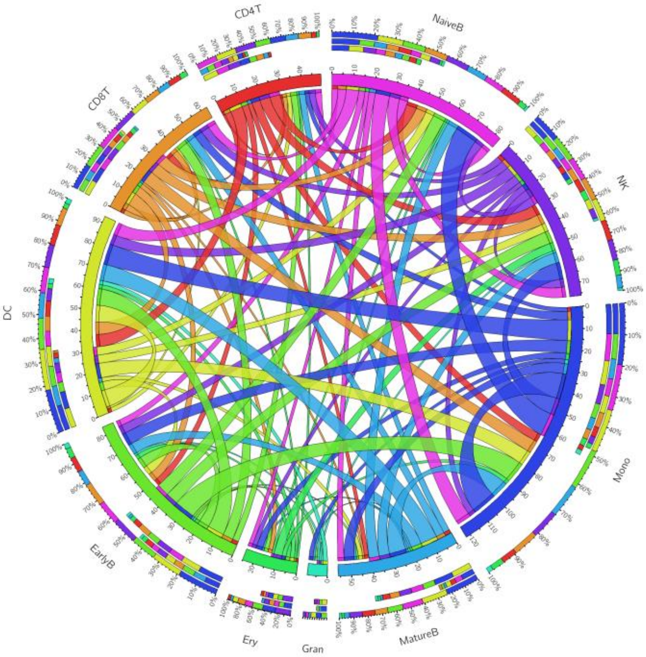

MGUS

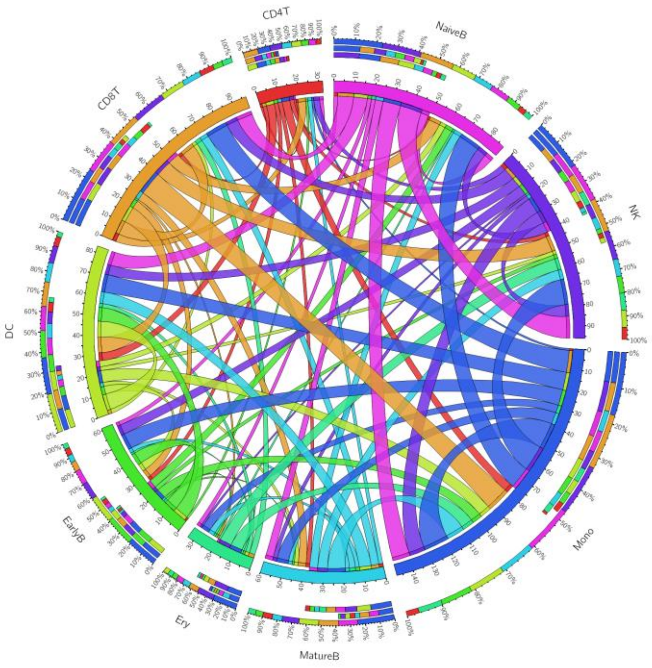

SMM

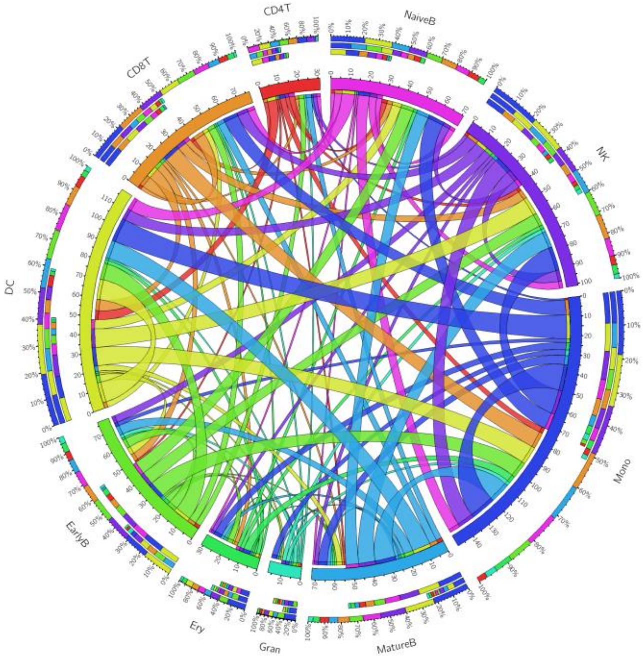

Figure S4. Circos plots of the CCEs in NBM and precursor stages of MM.

Figure S5.

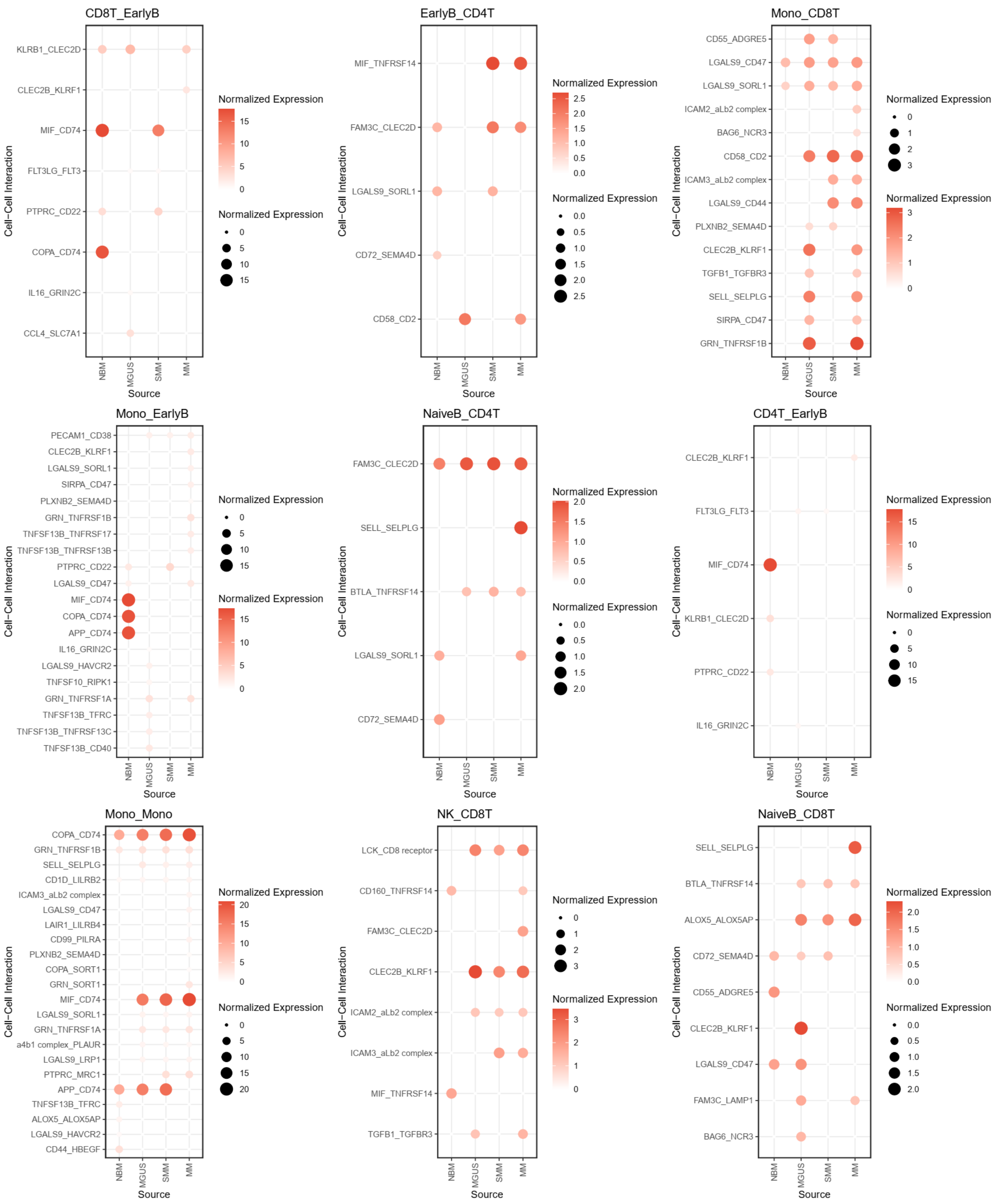

Figure S5. Dysregulated CCEs interacted in different cell types in the four stages.

Figure S6.

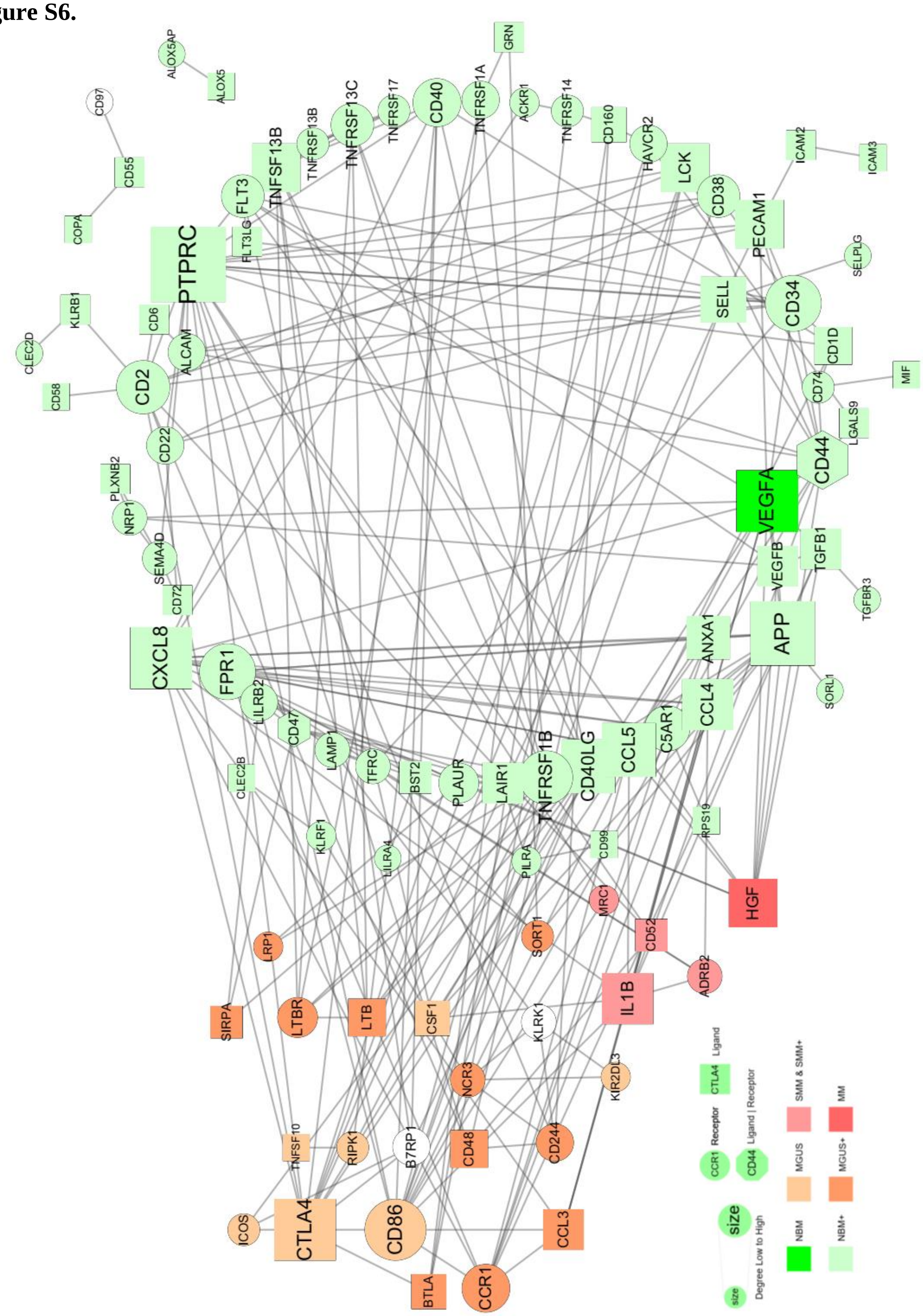

Figure S6. The PPI network constructed with ligand and receptor genes in CCEs.

Figure S7.

NBM

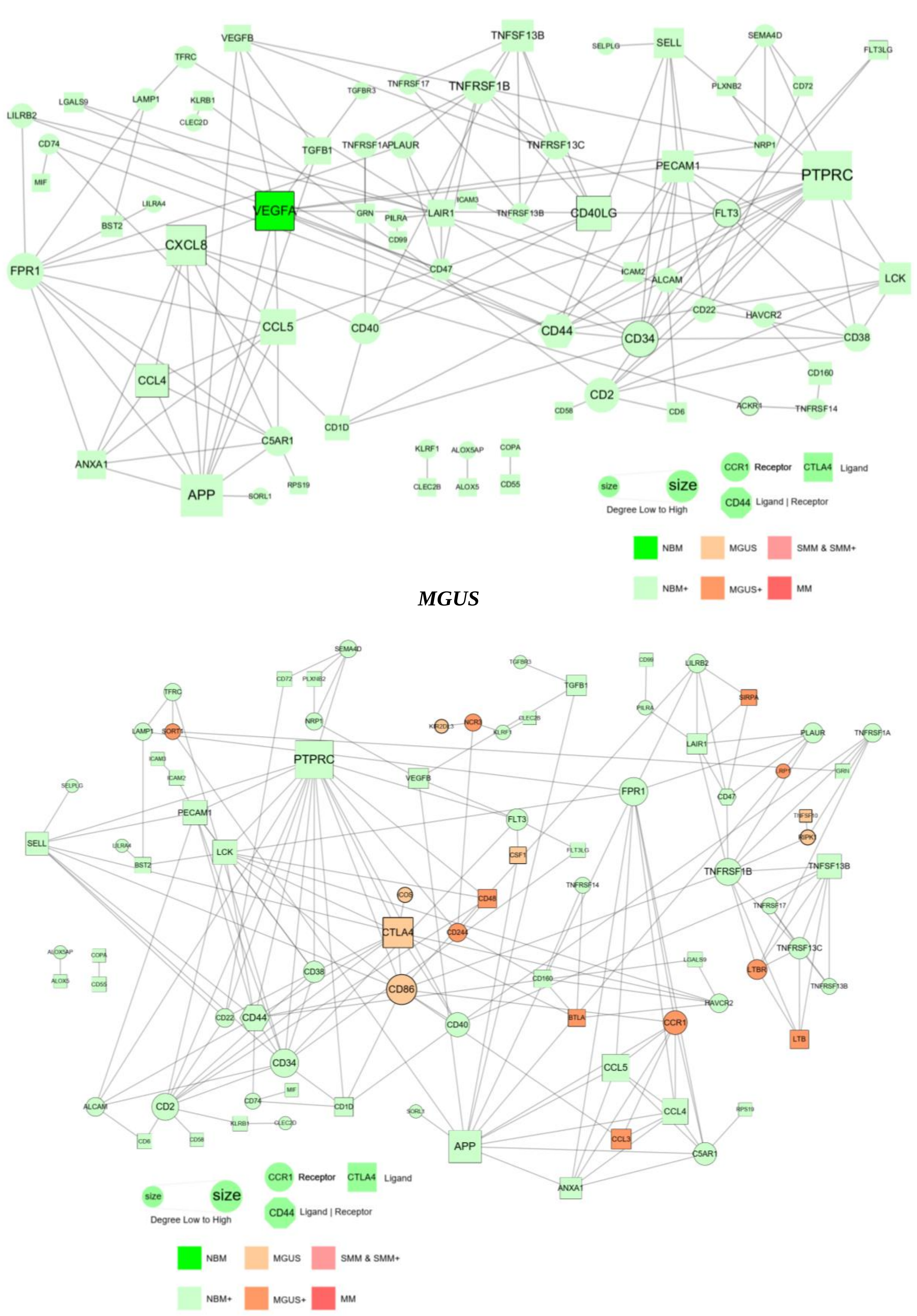

Figure S7. The PPI network constructed with ligand and receptor genes in CCEs in NBM and MGUS.

Figure S8.

SMM

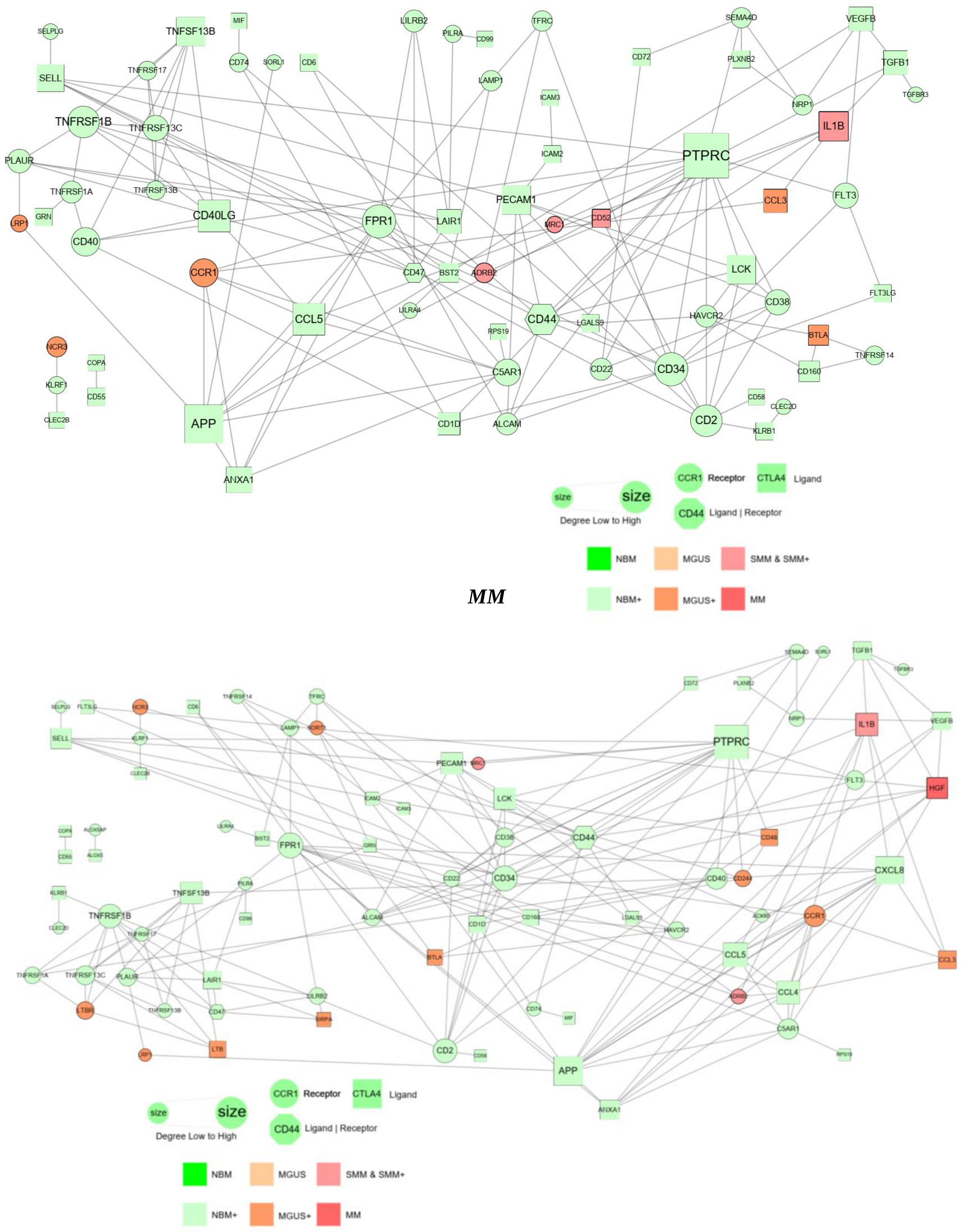

Figure S8. The PPI network constructed with ligand and receptor genes in CCEs in SMM and MM.

Figure S9.

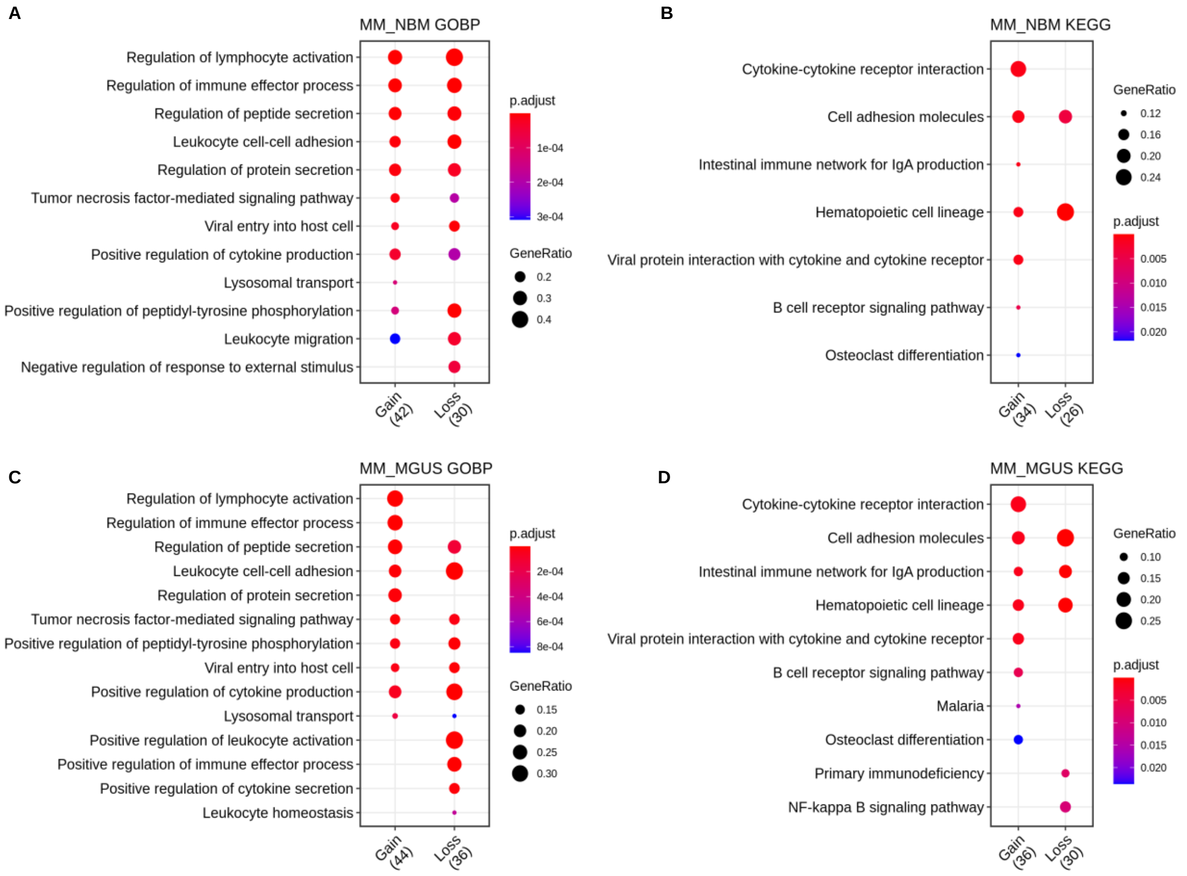

Figure S9. Functional enrichment analysis of the genes in stage specific networks. Enriched GO BP terms and KEGG pathways as Gain or Loss function in comparison of MM to NBM (A, B), and MM to MUGS (C, D).

Figure S10.

OS genes Functional enrichment analysis

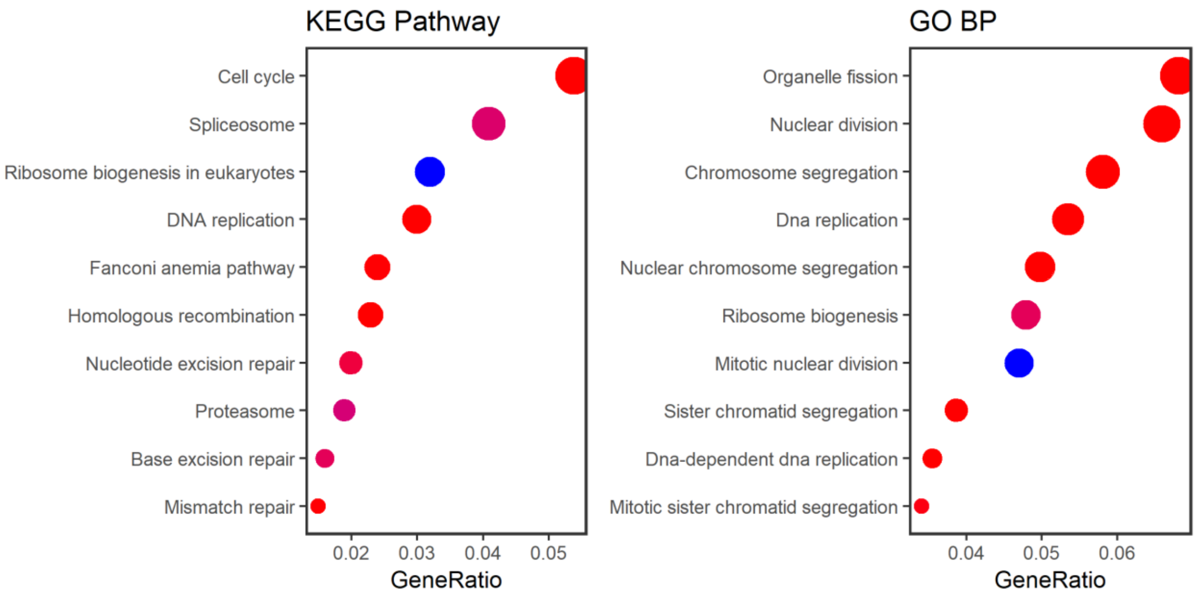

Figure S10. Enriched KEGG pathways and GO BP terms with MM OS related genes.

Figure S11.

ML in M KEGG and GO BP

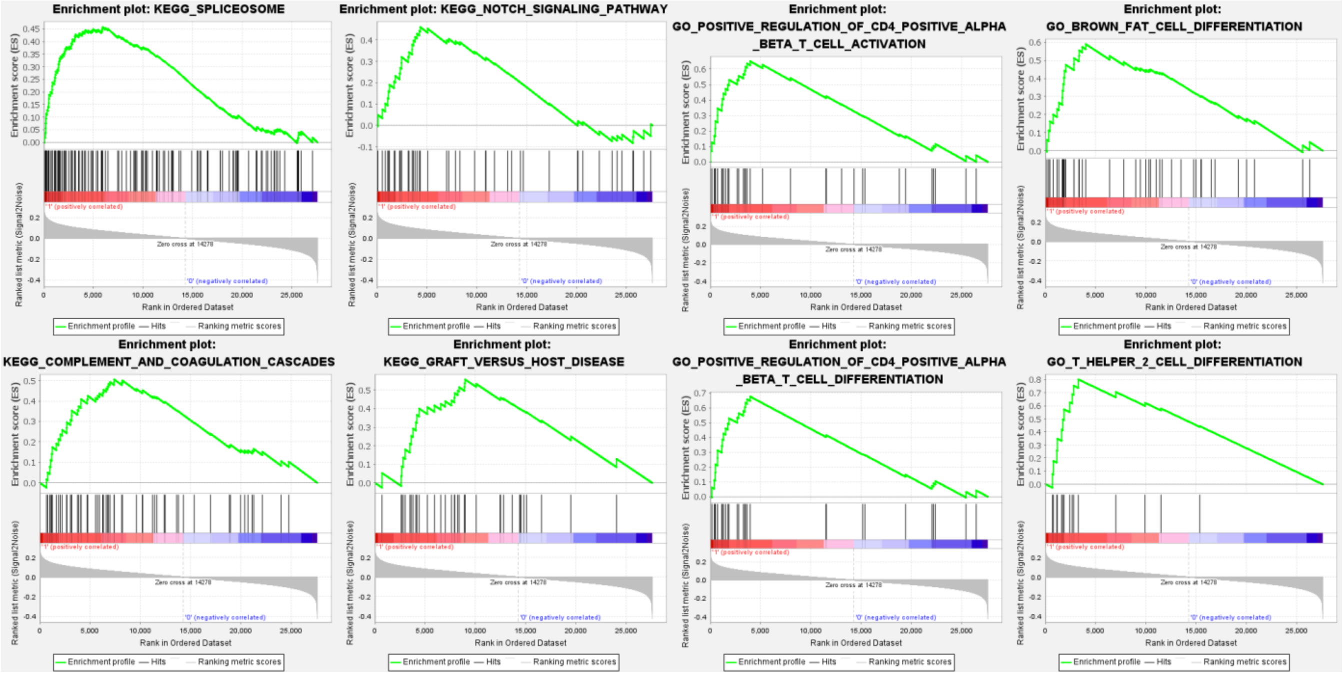

Figure S11. The results of GSEA analysis for comparison of medium risk group and low risk group.
